# Supplementary material for: Limited conservation in cross-species comparison of GLK transcription factor binding suggested wide-spread cistrome divergence
Source: Nat Commun. 2022 Dec 9;13:7632. doi: 10.1038/s41467-022-35438-4 (PMC9734178; doi:10.1038/s41467-022-35438-4)
Supplement: Supplementary file 1 — Supplementary Information [file 41467_2022_35438_MOESM1_ESM.pdf]

Limited conservation in cross-species comparison of GLK transcription factor binding suggested wide-spread cistrome divergence

Tu *et al.*

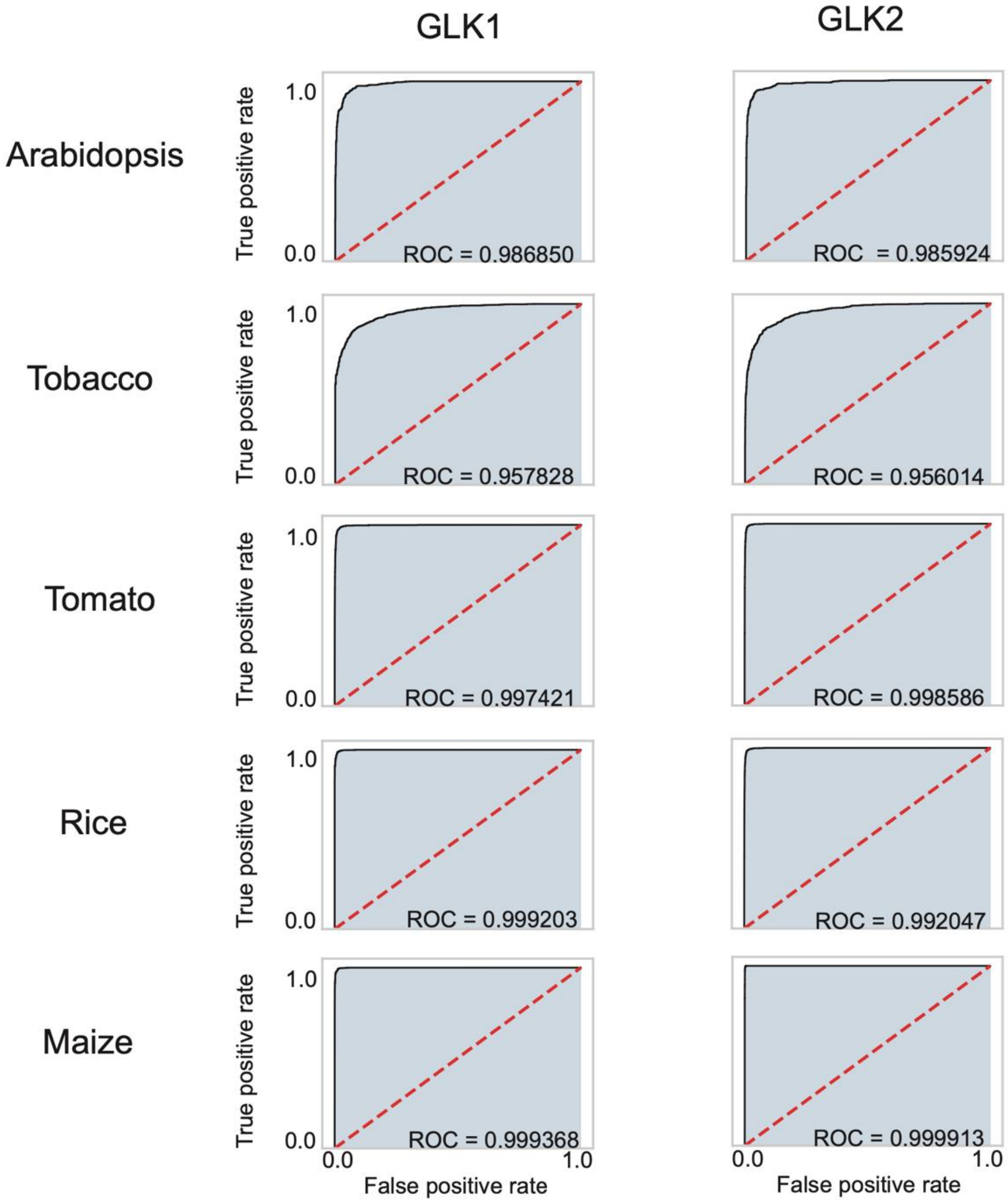

Supplementary Fig. 1. ROC curves for bag-of-*k*-mer models. The *k*-mer grammar tool was used to train models to distinguish GLK bound regions and the background. We tested both the 150bp and 300bp sequences centered at the peak summit for model training. The best results were obtained with 150 bp sequences in the Arabidopsis, tomato, rice and maize genome, while the 300bp peak yielded the best result for the tobacco models. The high area under the ROC curve value suggested that the *k*-mer models can learn the binding site sequence signatures and predict GLK binding with high the high accuracy and precision.

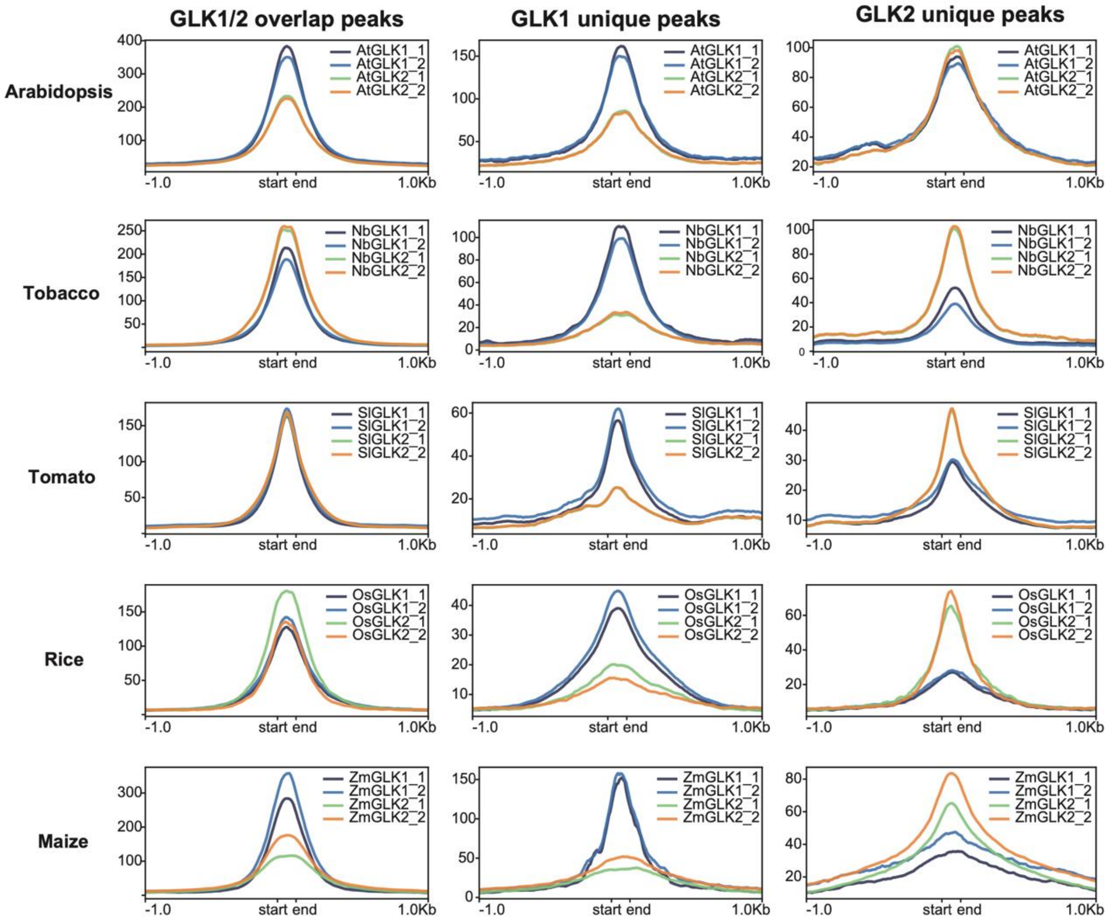

Supplementary Fig. 2. ChIP-seq signal in the GLK1 and GLK2 overlap and unique peaks. The average GLK1 and GLK2 ChIP-seq signals in the 1kb regions up- and down-stream of the peak summits were plotted. High ChIP-seq signal for both GLKs was observed in the overlap peaks. In GLK1 CHIP-seq peaks that were not called as GLK2 peaks, we could still identified weak GLK2 signal, and vise versa. Suggesting that those GLK1- or GLK2-species peaks are weaker than the overlap ones. ChIP-seq read counts are normalized to RPKM and then by FRiP. Source data are provided as a Source Data file.

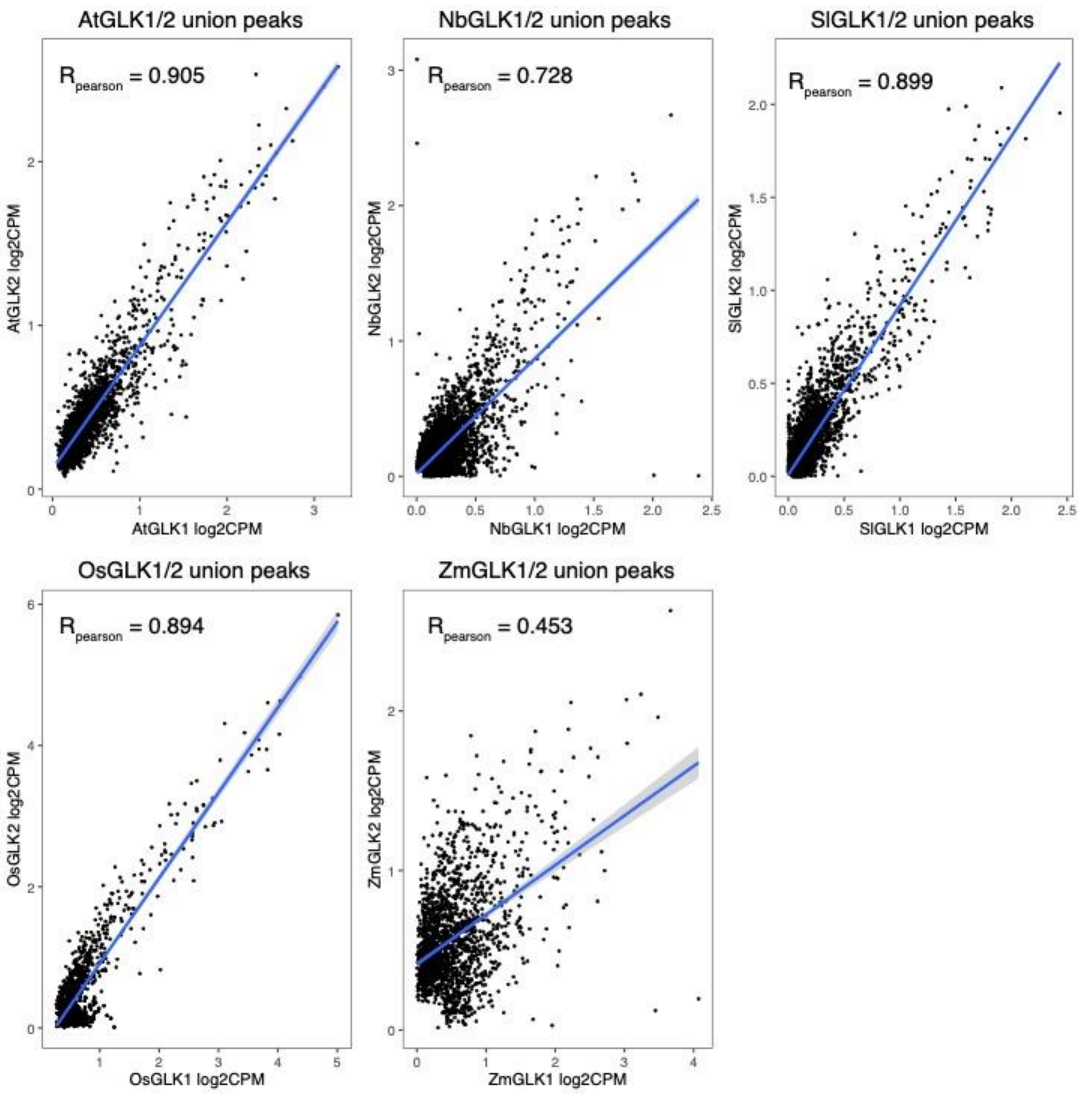

Supplementary Fig. 3. Compare GLK1 and GLK2 ChIP-seq signals in their ChIP-seq peak union. The GLK1 and GLK2 ChIP-seq peaks were merged. Scatter plots showed their read counts (log2CPM) in the region 250 bp up- and down-stream of the peak summit. The error band represent standard error calculated by ggplot2::geom_smooth. Source data are provided as a Source Data file.

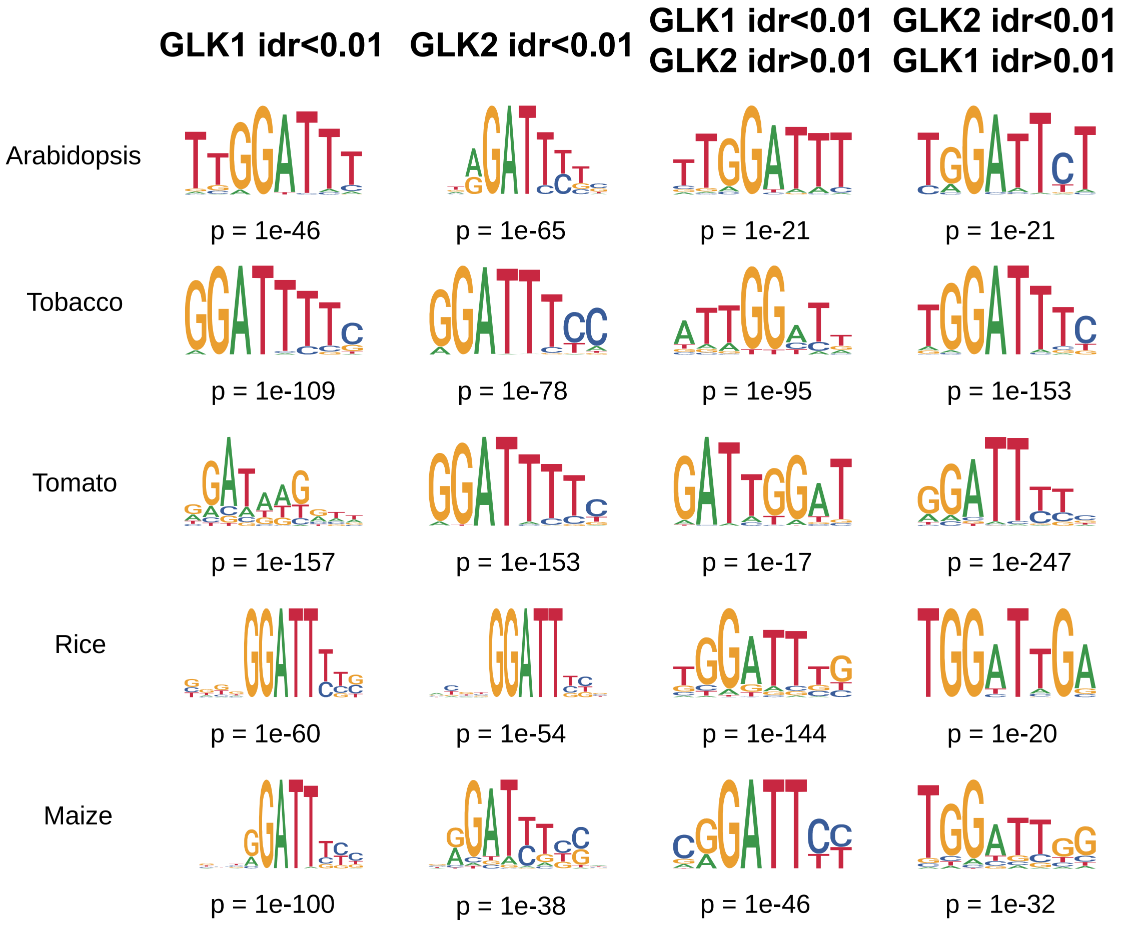

Supplementary Fig. 4. Motifs enrichment analysis of GLK1 and GLK2 overlap and unique peaks. The enriched motif in GLK1 and GLK2 overlap peaks (both GLK1 and GLK2 IDR <0.01) and unique peaks that didn't pass the IDR 0.01 cut off. All of them showed the same motif enrichment, suggesting those weak non-overlap peaks also have the GLK binding sites.

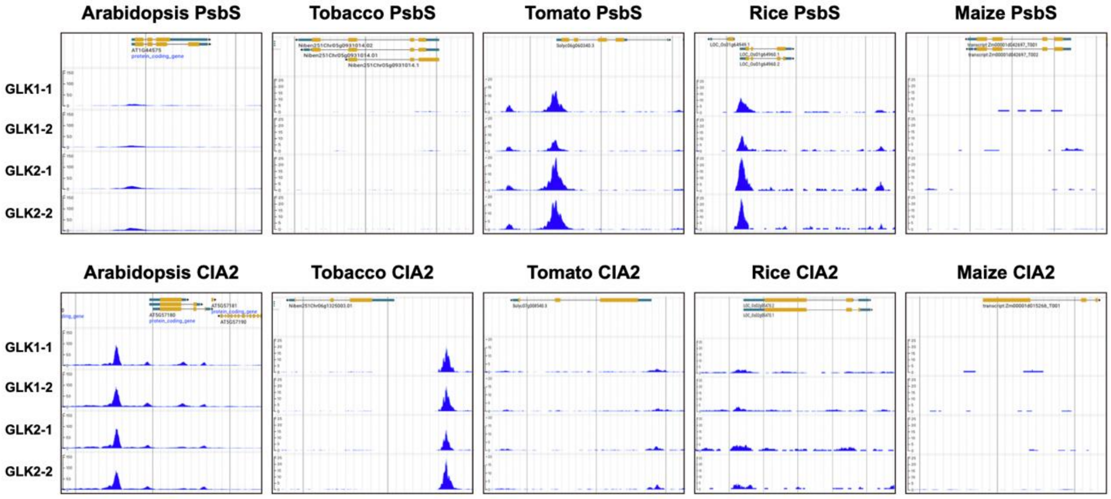

Supplementary Fig. 5. Species-specific GLK ChIP-seq target genes. Genome browser tracks showing GLK1/2 ChIP-seq reads in genes encoding photosystem II subunit S (PsbS) and CHLOROPLAST IMPORT APPARATUS 2 (CIA2). Only the tomato and rice *PsbS* genes were called as GLK ChIP-seq targets, while the *CIA2* genes in Arabidopsis and tobacco were called as GLK ChIP-seq targets. For within species comparison, the PsbS and CIA2 tracks of the same species used the same y-axis scale.

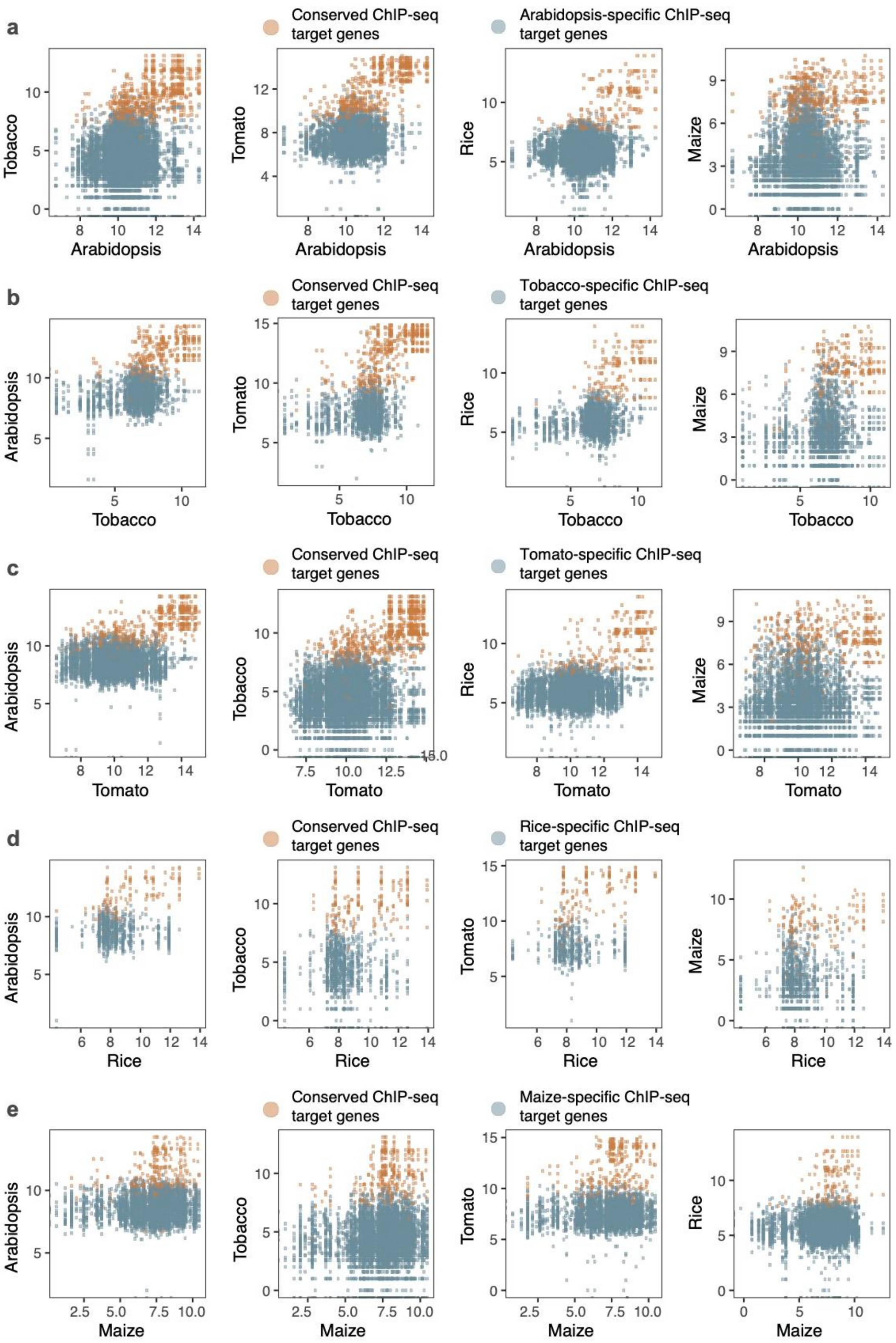

Supplementary Fig. 6. Compare promoter GLKs ChIP-seq coverage between species. Scatter plots showing the GLKs ChIP-seq read coverage of the conserved and species-specific target genes in each species. **a.** Arabidopsis. **b.** tobacco. **c.** tomato. **d.** rice. **e.** maize. The blue dots are a pair of orthologous genes with only one of them

bound by GLK in a two species comparison. The x-axis and y-axis are the GLKs ChIP-seq read counts (Log2CPM) of the gene pair in each species. For each pair-wise cross-species comparison, homologous gene pairs berween two species were called using blastp (evalue <1e-50). The combined GLK1 and GLK2 ChIP-seq reads were then counted in the proximal promoter regions (1kb upstream and -500 bp downstream of gene TSS) of the conserved and species-specific GLK target gene pairs. It is clear that the conserved ones have high ChIP-seq signal in both species, while the species-specific ones has lower signal in one species. Source data are provided as a Source Data file.

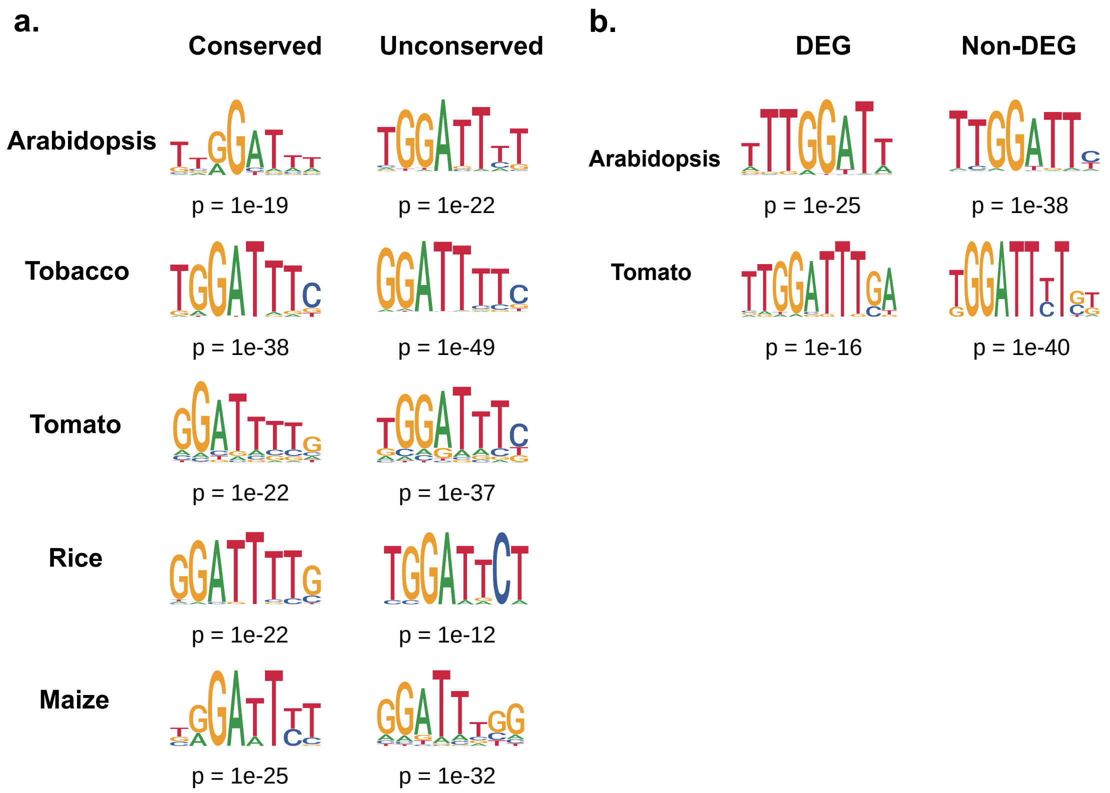

Supplementary Fig. 7. Motifs enrichment analysis of conserved and non-onserved GLK target genes, as well as GLK mutant DEG and non-DEGs. a. The GLK core binding motif GATT is enriched in both the species-conserved and the species-specific ChIP-seq target genes. **b.** Motifs identified in the tomato and Arabidopsis glk double mutant's differentially expressed and non-differentially expressed GLK target genes are also similar.

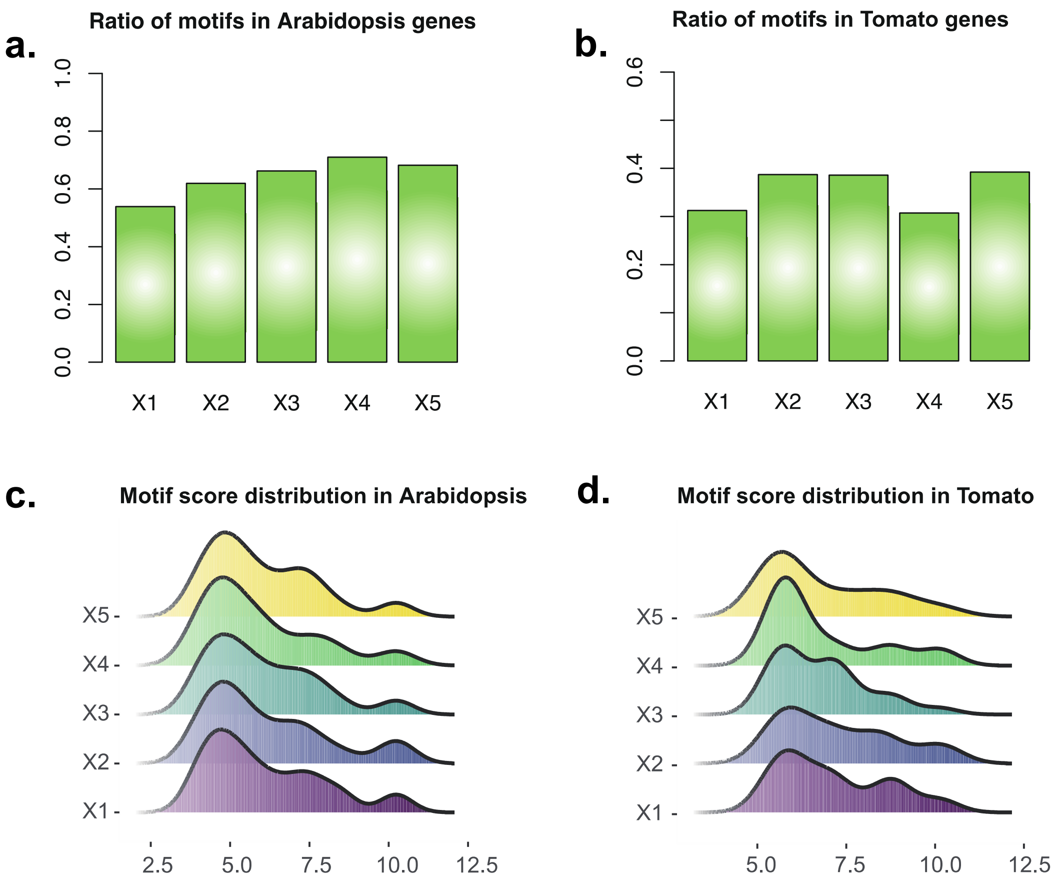

Supplementary Fig. 8. TFBS analysis of genes in five conservation groups. a. Percentage of genes contained GLK TF binding sites called by HOMER motif search in five Arabidopsis GLK ChIP-seq target genes conservation groups. X indicates the number of species that the gene is conserved in. **b.** Motif ratio in tomato GLK target genes found in leaf in the five conservation groups. **c.** HOMER motif hit score distribution in Arabidopsis. **d.** Motif score distribution in tomato. The results showed that the conserved and non-conserved genes have similar motif hit ratio and score. Source data are provided as a Source Data file.

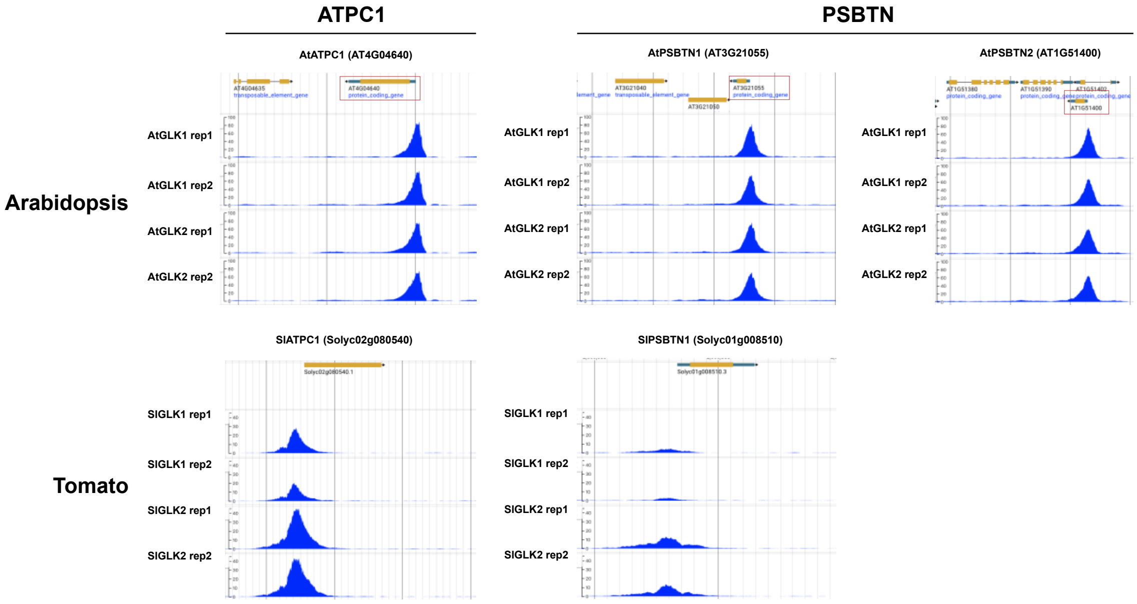

Supplementary Fig. 9. GLK binding sites in the ATPC1 and PSBTN genes. Despite the fact that ATPC1 has strong GLK binding sites in both Arabidopsis and tomato, it is only differentially expressed in tomato, suggesting other co-binding TF could compensate for the loss of GLK in Arabidopsis. On the other hand, the two Arabidopsis PSBTN genes have strong GLK binding sites. The tomato PSBTN has a weak GLK binding site. But PSBTN are differentially expressed in the GLK mutants in both species, suggesting that binding strength is not always correlated with transcription regulatory potential of the binding site.

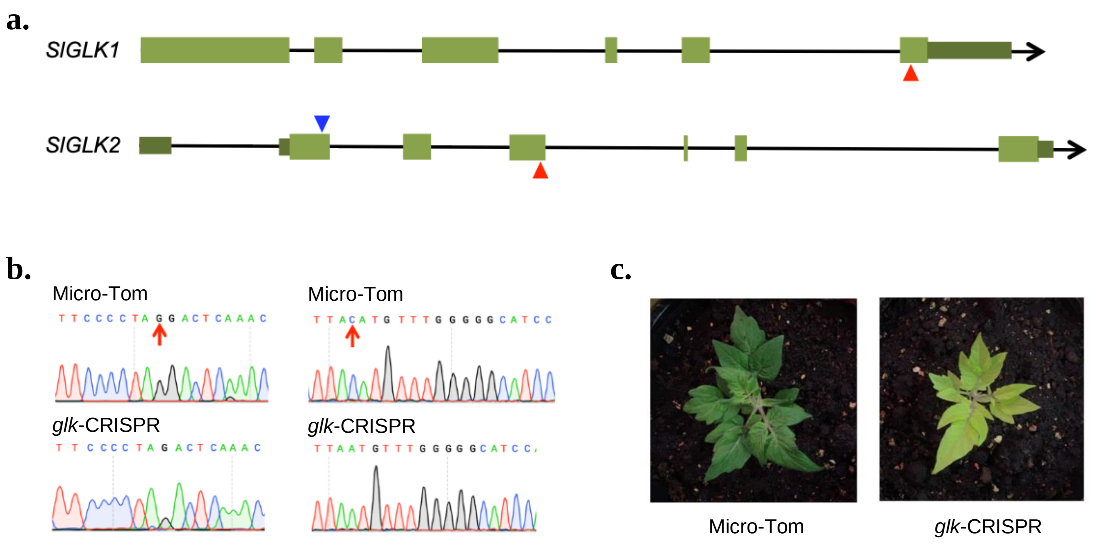

Supplementary Fig. 10. Characterization of the tomato GLK double mutant. a. Gene structure of GLK1 and GLK2 in tomato. The blue arrow indicates the GLK2 natural mutation site in the microTom background. The two red arrow indicate the CRISPR guide RNA target sites in GLK1 and GLK2. **b.** The deletions caused by CRISPR in GLK1 and GLK2 loci are confirmed by Sanger DNA sequencing. **c.** Pale-green phenotype of the double mutant and a wild-type microTom as control. Primers used for CRISPR gRNA: SlGLK-crF1(GCCGGAGGTTTGAGTCCTAGGTTTTAGAGCTAGAAATAGC), SlGLK-crR1(CTAGGACTCAAACCTCCGGCAAGCCTACTGGTTCGCTTGA); SlGLK-crF1(GGATGCCCCCAAACATGTAAGTTTTAGAGCTAGAAATAGC), SlGLK-crR2(TTACATGTTTGGGGGCATCCAAGCCTACTGGTTCGCTTGA). Primers used for genotyping: SlGLK1cr-gt-F(CATAGATGCAGCCATTGGAGATG), SlGLK1cr-gt-R(GCATTGCTAGCTGCCTCATTAAA); 2) SlGLK2cr-gt-F(AGCTGAAGCAGCGAGCTTGAAC),SlGLK2cr-gtR(CTTTGATGATGGCAGGGCTTGG).

Supplementary Table 1. GLK genes used for ChIP-seq.

**Species**	**Gene**	**ID**	**Epitope Tag**	**Plant material**	**Vector**
Arabidopsis	GLK1	AT2G20570	GFP	*glk1/glk2* (N9807)	pK7FWG2
Arabidopsis	GLK2	AT5G44190	GFP	*glk1/glk2* (N9807)	pK7FWG2
Tobacco	GLK1	Niben251Chr11g0028007	HA	*N benthamiana*	pGWB14
Tobacco	GLK2	Niben251Chr10g0202013	HA	*N benthamiana*	pGWB14
Tomato	GLK1	Solyc07g053630	GFP	*Solanum lycopersicum* AC++	pK7FWG2
Tomato	GLK2	Solyc10g008160	GFP	*Solanum lycopersicum* MicroTom	pK7FWG2
Rice	GLK1	LOC_Os06g24070	HA	Oryza sativa Nipponbare	pCAMBIA1301
Rice	GLK2	LOC_Os01g13740	HA	Oryza sativa Nipponbare	pCAMBIA1301
Maize	GLK1	Zm00001d044785	Biotin	B73 leaf protoplast (Tu et al. 2020)	p35S-GW-3Avi
Maize	GLK2	Zm00001d039260	Biotin	B73 leaf protoplast (Tu et al. 2020)	p35S-GW-3Avi

Supplementary Table 2. Different conserved target identified by the ENCODE2 spp-IDR-TIP ChIP-seq pipeline at high and low stringency.

**Species**	***p*-value cut-off**	**GLK targets**	**Conserved targets**	**% of conservation targets**
Arabidopsis	0.05	961	143	14.88%
Arabidopsis	0.01	495	69	13.94%
Tobacco	0.05	743	139	18.71%
Tobacco	0.01	608	87	14.31%
Tomato	0.05	837	136	16.25%
Tomato	0.01	600	77	12.83%
Rice	0.05	347	75	21.61%
Rice	0.01	252	52	20.63%
Maize	0.05	886	153	17.27%
Maize	0.01	593	92	15.51%

Supplementary Table 3. Conservation score of GLK target genes.

	**All GLK target genes**	**Photosynthesis genes targeted by GLK**	**Tetrapyrrole biosynthesis genes targeted by GLK**
**Arabidopsis**	0.475625	0.9538462	0.8545455
**Niben**	0.53159	0.9835616	0.7444444
**Tomato**	0.4578538	0.9333333	0.725
**Tobacco**	0.6108434	0.9555556	0.84
**Maize**	0.4552801	0.95625	0.88
