# Supplementary material for: Limited conservation in cross-species comparison of GLK transcription factor binding suggested wide-spread cistrome divergence
Source: Nat Commun. 2022 Dec 9;13:7632. doi: 10.1038/s41467-022-35438-4 (PMC9734178; doi:10.1038/s41467-022-35438-4)
Supplement: Supplementary file 3 — Description of Additional Supplementary Files [file 41467_2022_35438_MOESM3_ESM.pdf]

Description of Additional Supplementary Files

File Name: Supplementary Data 1
Description: ENCODE2 QC matrix for the ChIP-seq libraries

File Name: Supplementary Data 2
Description: GLK target genes in Arabidopsis. The differentially expressed *p*-val is calculated by DESEQ2 using Wald test and the adjusted *p*-val is calculated using the Benjamini-Hochberg method.

File Name: Supplementary Data 3
Description: GLK target genes in tobacco.

File Name: Supplementary Data 4
Description: GLK target genes in tomato. The differentially expressed *p*-val is calculated by DESEQ2 using Wald test and the adjusted *p*-val is calculated using the Benjamini-Hochberg method

File Name: Supplementary Data 5
Description: GLK target genes in rice

File Name: Supplementary Data 6
Description: GLK target genes in maize

File Name: Supplementary Data 7
Description: GLK motif enrichment analysis in ChIP-seq peaks

File Name: Supplementary Data 8
Description: *K*-mer model statistics

File Name: Supplementary Data 9
Description: Top 10 *k*-mers in *k*-mer models

File Name: Supplementary Data 10
Description: List of genes involved in the chloroplast photosynthetic electron transfer chain

File Name: Supplementary Data 11
Description: Orthologous GLK targets in the chlorophyll biosynthesis pathways and their GLK ChIP-seq signal

File Name: Supplementary Data 12
Description: Differentially expressed genes in Arabidopsis glk1/glk2 mutant. The differentially expressed *p*-val is calculated by DESEQ2 using Wald test and the adjusted *p*-val is calculated using the Benjamini-Hochberg method.

File Name: Supplementary Data 13
Description: Differentially expressed genes in the leaf tissue of tomato glk1/2 double mutant.The differentially expressed *p*-val is calculated by DESEQ2 using Wald test and the adjusted *p*-val is calculated using the Benjamini-Hochberg method.
