# Supplementary material for: Limited conservation in cross-species comparison of GLK transcription factor binding suggested wide-spread cistrome divergence
Source: Nat Commun. 2022 Dec 9;13:7632. doi: 10.1038/s41467-022-35438-4 (PMC9734178; doi:10.1038/s41467-022-35438-4)
Supplement: Supplementary file 5 — Reporting Summary [file 41467_2022_35438_MOESM5_ESM.pdf]

nature research

Corresponding author(s): Silin Zhong

Last updated by author(s): 28/11/22

Reporting Summary

Nature Research wishes to improve the reproducibility of the work that we publish. This form provides structure for consistency and transparency in reporting. For further information on Nature Research policies, see our Editorial Policies and the Editorial Policy Checklist.

Statistics

For all statistical analyses, confirm that the following items are present in the figure legend, table legend, main text, or Methods section.

n/a	Confirmed	
☐	☒	The exact sample size (*n*) for each experimental group/condition, given as a discrete number and unit of measurement
☐	☒	A statement on whether measurements were taken from distinct samples or whether the same sample was measured repeatedly
☐	☒	The statistical test(s) used AND whether they are one- or two-sided<br>*Only common tests should be described solely by name; describe more complex techniques in the Methods section.*
☒	☐	A description of all covariates tested
☐	☒	A description of any assumptions or corrections, such as tests of normality and adjustment for multiple comparisons
☐	☒	A full description of the statistical parameters including central tendency (e.g. means) or other basic estimates (e.g. regression coefficient) AND variation (e.g. standard deviation) or associated estimates of uncertainty (e.g. confidence intervals)
☐	☒	For null hypothesis testing, the test statistic (e.g. *F*, *t*, *r*) with confidence intervals, effect sizes, degrees of freedom and *P* value noted<br>*Give P values as exact values whenever suitable.*
☒	☐	For Bayesian analysis, information on the choice of priors and Markov chain Monte Carlo settings
☒	☐	For hierarchical and complex designs, identification of the appropriate level for tests and full reporting of outcomes
☐	☒	Estimates of effect sizes (e.g. Cohen's *d*, Pearson's *r*), indicating how they were calculated

Our web collection on statistics for biologists contains articles on many of the points above.

Software and code

Policy information about availability of computer code

Data collection	No software was used for data collection.
Data analysis	Preprocessing of ChIP-seq and RNA-seq were performed using Bowtie 2 (version 2.3.2), SAMtools (version 1.9), deepTools (version 3.5.1), MACS2 (version 2.2.1),<br>IDR (version 2.0.4.2), PhantomPeakQualTools (version 1.14), HISAT2 (version 2.1.0), HTSeq (version 0.11.0) and DESeq2.<br>Function and motif enrichment analysis were used OrthoFinder (version 2.2.7), agriGO (version 2.0), MAPMAN (version 3.6.0) and HOMOER (version 4.11).<br>The synteny analysis and ks calculation were performed by MCScanX and KaKs_Calculator (version 2.0).<br>The code of machine learning analyses and downstream data analysis have been deposited to GitHub: https://github.com/rensabella/GLK-project.

For manuscripts utilizing custom algorithms or software that are central to the research but not yet described in published literature, software must be made available to editors and reviewers. We strongly encourage code deposition in a community repository (e.g. GitHub). See the Nature Research guidelines for submitting code & software for further information.

Data

Policy information about availability of data

All manuscripts must include a data availability statement. This statement should provide the following information, where applicable:

- Accession codes, unique identifiers, or web links for publicly available datasets
- A list of figures that have associated raw data
- A description of any restrictions on data availability

The data generated in this study gave been deposited in the NCBI database under accession code PRJNA682315 (https://www.ncbi.nlm.nih.gov/bioproject/PRJNA682315) and GitHub (https://github.com/rensabella/GLK-project/tree/main/data). Source data are provided with this paper. Published data used in the study are from PRJNA518749 (SRR8525119, SRR8525138, SRR8525003 and SRR8525004) and PRJNA743574 (SRR15036576, SRR15036577, SRR15036615 and SRR15036616). The Arabidopsis TF co-binding data is from the plant cistrome database (http://neomorph.salk.edu/dap_web/pages/index.php). The Arabidopsis nucleotide diversity data is from the 1001 Genomes database (https://1001genomes.org/data/GMI-MPI/releases/v3.1/). The maize 23 tissue gene expression data is from maizeGDB (https://www.maizegdb.org/expression).

Field-specific reporting

Please select the one below that is the best fit for your research. If you are not sure, read the appropriate sections before making your selection.

☒ Life sciences ☐ Behavioural & social sciences ☐ Ecological, evolutionary & environmental sciences

For a reference copy of the document with all sections, see nature.com/documents/nr-reporting-summary-flat.pdf

Life sciences study design

All studies must disclose on these points even when the disclosure is negative.

Sample size	No sample size calculation was performed.
Data exclusions	No data exclusion in the study.
Replication	Two replicates for ChIP-seq. Three replicates for RNA-seq samples.
Randomization	For all experiments, treatment and control samples were grown side by side, each replicate were performed on separate plant.
Blinding	No blinding used.

Reporting for specific materials, systems and methods

We require information from authors about some types of materials, experimental systems and methods used in many studies. Here, indicate whether each material, system or method listed is relevant to your study. If you are not sure if a list item applies to your research, read the appropriate section before selecting a response.

Materials & experimental systems

n/a	Involved in the study	
☐	☒	Antibodies
☒	☐	Eukaryotic cell lines
☒	☐	Palaeontology and archaeology
☒	☐	Animals and other organisms
☒	☐	Human research participants
☒	☐	Clinical data
☒	☐	Dual use research of concern

Methods

n/a	Involved in the study	
☐	☒	ChIP-seq
☒	☐	Flow cytometry
☒	☐	MRI-based neuroimaging

Antibodies

Antibodies used	Anti-GFP (#A-11122, ThermoFisher) and anti-HA (#C29F4, Cell Signaling Technology)
Validation	Anti-GFP (#A-11122, ThermoFisher):<br>(https://www.thermofisher.com/antibody/product/GFP-Antibody-Polyclonal/A-11122)<br>Anti-HA (#C29F4, Cell Signaling Technology):<br>(https://www.cellsignal.com/products/primary-antibodies/ha-tag-c29f4-rabbit-mab/3724)

ChIP-seq

Data deposition

☒ Confirm that both raw and final processed data have been deposited in a public database such as GEO.

☒ Confirm that you have deposited or provided access to graph files (e.g. BED files) for the called peaks.

Data access links
May remain private before publication.

Sequencing data have been deposited in the NCBI SRA database under the accession number PRJNA682315 (https://www.ncbi.nlm.nih.gov/bioproject/PRJNA682315). The processed data generated are available in GitHub (https://github.com/rensabella/GLK-project).

Files in database submission

Raw data:
SRR13189558_1.fastq.gz
SRR13189558_2.fastq.gz
SRR13189559_1.fastq.gz
SRR13189559_2.fastq.gz
SRR13189560_1.fastq.gz
SRR13189560_2.fastq.gz
SRR13189561_1.fastq.gz
SRR13189561_2.fastq.gz
SRR13189562_1.fastq.gz
SRR13189562_2.fastq.gz
SRR13189563_1.fastq.gz
SRR13189563_2.fastq.gz
SRR13189564_1.fastq.gz
SRR13189564_2.fastq.gz
SRR13189569_1.fastq.gz
SRR13189569_2.fastq.gz
SRR13189579_1.fastq.gz
SRR13189579_2.fastq.gz
SRR13189580_1.fastq.gz
SRR13189580_2.fastq.gz
SRR13189581_1.fastq.gz
SRR13189581_2.fastq.gz
SRR13189582_1.fastq.gz
SRR13189582_2.fastq.gz
SRR15036576_1.fastq.gz
SRR15036576_2.fastq.gz
SRR15036577_1.fastq.gz
SRR15036577_2.fastq.gz
SRR15036615_1.fastq.gz
SRR15036615_2.fastq.gz
SRR15036616_1.fastq.gz
SRR15036616_2.fastq.gz
SRR8525003_1.fastq.gz
SRR8525003_2.fastq.gz
SRR8525004_1.fastq.gz
SRR8525004_2.fastq.gz
SRR8525119_1.fastq.gz
SRR8525119_2.fastq.gz
SRR8525138_1.fastq.gz
SRR8525138_2.fastq.gz

Bigwig files:
SRR13189558.bw
SRR13189559.bw
SRR13189560_rmdup.bw
SRR13189561_rmdup.bw
SRR13189562_rmdup.bw
SRR13189563_rmdup.bw
SRR13189564_rmdup.bw
SRR13189569_rmdup.bw
SRR13189579.bw
SRR13189580.bw
SRR13189581_rmdup.bw
SRR13189582_rmdup.bw
SRR15036576_rmdup.bw
SRR15036577_rmdup.bw
SRR15036615_rmdup.bw
SRR15036616_rmdup.bw
SRR8525003.bw
SRR8525004.bw
SRR8525119.bw

SRR8525138.bw

Peak files:
SRR13189558_sub_peaks.narrowPeak
SRR13189559_sub_peaks.narrowPeak
SRR13189560_sub_peaks.narrowPeak
SRR13189561_sub_peaks.narrowPeak
SRR13189562_sub_peaks.narrowPeak
SRR13189563_sub_peaks.narrowPeak
SRR13189564_sub_peaks.narrowPeak
SRR13189569_sub_peaks.narrowPeak
SRR13189579_sub_peaks.narrowPeak
SRR13189580_sub_peaks.narrowPeak
SRR13189581_sub_peaks.narrowPeak
SRR13189582_sub_peaks.narrowPeak
SRR15036576_sub_peaks.narrowPeak
SRR15036577_sub_peaks.narrowPeak
SRR15036615_sub_peaks.narrowPeak
SRR15036616_sub_peaks.narrowPeak
SRR8525003_sub_peaks.narrowPeak
SRR8525004_sub_peaks.narrowPeak
SRR8525119_sub_peaks.narrowPeak
SRR8525138_sub_peaks.narrowPeak

Genome browser session (e.g. UCSC)

http://www.epigenome.cuhk.edu.hk/jbrowse2/

Methodology

Replicates

For the five species' GLK1 and GLK2 ChIP-seq peak calling, two replicates were used.

Sequencing depth

5-20 million reads per sample were obtained for Arabidopsis ChIP-seq;PE;
20-30 million reads per sample were obtained for tomato ChIP-seq; PE;
10-15 million reads per sample were obtained for tabacco ChIP-seq; PE;
4-8 million reads per sample were obtained for rice ChIP-seq; PE;
30-40 million reads per sample were obtained for maize ChIP-seq; PE;
20-40 million reads per sample were obtained for Arabidopsis RNA-seq; PE;
20-40 million reads per sample were obtained for tomato RNA-seq; PE.

Antibodies

Anti-GFP (#A-11122, ThermoFisher) and anti-HA (#C29F4, Cell Signaling Technology)

Peak calling parameters

macs2 callpeak --call-summits -f BAMPE -g “effective genome size” -t -c
idr --samples --input-file-type narrowPeak --rank signal.value --idr-threshold 0.01 --output-file

Data quality

Replicate1 NSC1 RSC1 FRiP1 Replicate2 NSC2 RSC2 FRiP2 Correlation
at_g1_rep1 1.12 1.26 0.3718062 at_g1_rep2 1.1 1.25 0.3565357 0.9952
at_g2_rep1 1.09 1.27 0.4294187 at_g2_rep2 1.08 1.27 0.4243385 0.9951
nben_g1_rep1 1.73 1.17 0.1142368 nbent_g1_rep2 1.83 1.2 0.1158178 0.9976
nben_g2_rep1 1.77 1.39 0.06215453 nben_g2_rep2 2 1.69 0.03483229 0.8912
sl_g1_rep1 1.25 1.08 0.2258678 sl_g1_rep2 1.16 1.1 0.1723397 0.9877
sl_g2_rep1 1.35 1.06 0.2991488 sl_g2_rep2 1.38 1.06 0.3008416 0.9987
sl_fruit_g1_rep1 1.21 1.18 0.4248145 sl_fruit_g1_rep2 1.22 1.17 0.4283253 0.9985
sl_fruit_g2_rep1 1.21 1.23 0.3006065 sl_fruit_g2_rep2 1.21 1.2 0.3014485 0.9981
rice_g1_rep1 1.17 1.17 0.1886353 rice_g1_rep2 1.17 1.23 0.1873289 0.9957
rice_g2_rep1 1.4 1.13 0.08994993 rice_g2_rep2 1.3 1.18 0.0562373 0.9744
zm_g1_rep1 1.3 1.6 0.07563139 zm_g1_rep2 1.94 1.06 0.1370594 0.8804
zm_g2_rep1 2.57 1.11 0.247976 zm_g2_rep2 1.31 1.08 0.06968715 0.9498
(Also in Supplementary Data 2)

Software

Bowtie 2 (version 2.3.2),
SAMtools (version 1.9),
deepTools (version 3.5.1),
MACS2 (version 2.2.1),
IDR (version 2.0.4.2),
PhantomPeakQualTools (version 1.14),
HISAT2 (version 2.1.0),
HTSeq (version 0.11.0),
DESeq2,
agriGO (version 2.0),
MAPMAN (version 3.6.0),
HOMOER (version 4.11),
OrthoFinder (version 2.2.7),
MCScanX,

KaKs_Calculator (version 2.0)
